# A Web-Based Self-Titration Program to Control Blood Pressure in Patients With Primary Hypertension: Randomized Controlled Trial

**DOI:** 10.2196/15836

**Published:** 2019-12-05

**Authors:** Chi-Wen Kao, Ting-Yu Chen, Shu-Meng Cheng, Wei-Shiang Lin, Yue-Cune Chang

**Affiliations:** 1 Department of Nursing Tri-Service General Hospital Taipei Taiwan; 2 School of Nursing National Defense Medical Center Taipei Taiwan; 3 School of Nursing Chung-Jen Junior College of Nursing, Health Sciences and Management Chiayi Taiwan; 4 Division of Cardiology Department of Internal Medicine Tri-Service General Hospital Taipei Taiwan; 5 School of Medicine National Defense Medical Center Taipei Taiwan; 6 Division of Cardiology Tri-Service General Hospital Taipei Taiwan; 7 Department of Mathematics Tamkang University Taipei Taiwan

**Keywords:** Web-based, self-titration, blood pressure, hypertension, health-related quality of life

## Abstract

**Background:**

Hypertension is a major cause of mortality in cardiac, vascular, and renal disease. Effective control of elevated blood pressure has been shown to reduce target organ damage. A Web-based self-titration program may empower patients to control their own disease, share decisions about antihypertensive dose titration, and improve self-management, ultimately improving health-related quality of life.

**Objective:**

Our primary aim was to evaluate the effects of a Web-based self-titration program for improving blood pressure control in patients with primary hypertension. Our secondary aim was to evaluate the effects of that program on improving health-related quality of life.

**Methods:**

This was a parallel-group, double-blind, randomized controlled trial with assessments at baseline, 3 months, and 6 months. We included patients with primary hypertension (blood pressure>130/80 mm Hg) from a cardiology outpatient department in northern Taiwan and divided them randomly into intervention and control groups. The intervention group received the Web-based self-titration program, while the control group received usual care. The random allocation was concealed from participants and outcome evaluators. Health-related quality of life was measured by the EuroQol five-dimension self-report questionnaire. We used generalized estimating equations to evaluate the effects of the intervention.

**Results:**

We included 222 patients and divided them equally into intervention (n=111) and control (n=111) groups. Patients receiving the Web-based self-titration program showed significantly greater improvement in the systolic and diastolic blood pressure control than those who did not receive this program, at 3 months (–21.4 mm Hg and –5.4 mm Hg, respectively; *P*<.001) and 6 months (–27.8 mm Hg and –9.7 mm Hg, respectively; *P*<.001). Compared with the control group, the intervention group showed a significant decrease in the overall defined daily dose at both 3 (–0.202, *P*=.003) and 6 (–0.236, *P*=.001) months. Finally, health-related quality of life improved significantly in the intervention group compared with the control group at both 3 and 6 months (both, *P*<.001).

**Conclusions:**

A Web-based self-titration program can provide immediate feedback to patients about how to control their blood pressure and manage their disease at home. This program not only decreases mean blood pressure but also increases health-related quality of life in patients with primary hypertension.

**Trial Registration:**

ClinicalTrials.gov NCT03470974; https://clinicaltrials.gov/ct2/show/NCT03470974

## Introduction

The World Health Organization (WHO) reported that hypertension is one of the most common health concerns [1], with an estimated worldwide prevalence of more than 1.3 billion people [2]. The WHO also predicted that hypertension will affect 29% of the world’s population by 2025 [3]. In the United States, just about 49% of male patients and 55% of female patients had controlled hypertension between 2009 and 2012 [4]. Similarly, around 24% of the adult population in Taiwan (4.6 million people) had hypertension, and only 58% of hypertension patients had good control [5].

Hypertension is a major cause of mortality, resulting in 10.5 million deaths worldwide annually [6,7]. Death is a consequence of target organ damage due to hypertension, including cardiovascular, cerebrovascular, and kidney diseases [8-10]. In Asia, high systolic blood pressure is the main risk factor for stroke [11]. Hypertension and its comorbidity also affect health-related quality of life (HRQoL) [12]. A meta-analysis of 20 cross-sectional studies showed that patients with hypertension had poor HRQoL compared with normotensive adults [13]. Similar findings were reported from a meta-analysis of 21 observational studies in China [14].

The poor adherence to health behaviors, a significant barrier to optimal blood pressure control [15], is due to treatment regimen complexity, side effects of medicines, psychosocial distress, and poor health literacy [16,17]. Home blood pressure self-monitoring has been shown to improve blood pressure control [18,19] and help health care providers make appropriate treatment decisions [19]. A recent meta-analysis concluded that clinical interventions should be developed to help patients perform accurate self-monitoring of their blood pressure in order to achieve better blood pressure control [20]. Telemedicine could be useful in this regard because it not only allows patients to easily perform self-monitoring at home but also enables clinical professionals to have access to the data and give their feedback to patients immediately [21].

In clinical practice, a cardiologist usually starts antihypertensive therapy by medication titration based on a patient’s response and educates patients about self-monitoring and self-adjustment of dosages at home [22]. However, most patients are anxious about how to follow the complicated treatment regimen [22]. Medication titration strategies have been designed to successfully assist patients in self-titration of antidiabetic, oral anticoagulation, and beta-adrenergic receptor blocking medicines [23-25]. The titration process allows patients to perceive how their efforts improve their disease outcomes [26].

Few randomized controlled trials have investigated the outcomes of medication titration on blood pressure control in patients with hypertension. The Telemonitoring and Self-Management in Hypertension 2 (TASMINH2) trial combined telemonitoring with a titration strategy to improve blood pressure control for patients with hypertension [27]. The intervention group had significantly greater decreases in systolic blood pressure (SBP) and diastolic blood pressure (DBP)—3.7 mm Hg (95% CI 0.8-6.6) and 1.3 mm Hg (95% CI 0.3-2.6), respectively—compared with a control group after 12 months. In a second trial, Targets and Self-Management for the Control of Blood Pressure in Stroke and at Risk Groups (TASMIN-SR) [28], patients with secondary hypertension received training in self-titration of antihypertensives. Again, the results showed that patients in the intervention group had significant decreases in SBP (–8.8 mm Hg; 95% CI 4.9-12.7) and DBP (–3.1 mm Hg; 95% CI 0.7-5.5) after 12 months. The TASMINH4 trial evaluated the effectiveness of general practitioner–led titration of antihypertensives with either self-monitoring or self-monitoring plus telemonitoring on blood pressure control [29]. The trial showed that SBP significantly decreased after 12 months in both intervention groups, with differences of –3.5 mm Hg (95% CI –5.8 to –1.2) in the self-monitoring group and –4.7 mm Hg (95% CI –7.0 to –2.4) in the telemonitoring group. Moreover, DBP also decreased in both the self-monitoring group (–1.5 mm Hg, 95% CI −2.7 to −0.2) and the telemonitoring group (−1.3 mm Hg, 95% CI −2.5 to −0.02).

Nevertheless, two previous studies were unable to detect a significant finding of the effects of antihypertensive titration on blood pressure control [30,31]. The first, a two-armed trial, indicated that health coaching with home titration was not superior to usual care for blood pressure control after 6 months [30]. A second study, the Diabetes Risk Evaluation and Microalbuminuria 3 (DREAM 3) trial, assessed the effects of a nurse-lead titration strategy in patients with hypertension, diabetes, and microalbuminuria, but found no significant effect on either SBP or DBP control at 6 and 12 months [31]. Thus, there is no consistent evidence about the efficacy of medication titration on blood pressure control, and there is a notable lack of research data from Asia.

We aimed to evaluate the effectiveness of a Web-based self-titration program on blood pressure control in patients with primary hypertension. Our primary hypothesis was that patients receiving the Web-based self-titration program in the intervention group would have a better control of SBP and DBP than the control group after 3 and 6 months. The secondary hypothesis was that the patients in the intervention group would show greater improvement in HRQoL than those in the control group.

## Methods

### Study Design

This was a parallel-group, double-blind, randomized controlled trial. Participants were randomly assigned to an intervention group or a control group using a permuted block randomization design with a block size of 4. The random allocation was concealed from participants and outcome evaluators via the use of sequentially numbered opaque envelopes. Data were collected at baseline, 3 months, and 6 months. The study was based on the CONSORT-EHEALTH guidelines (V1.6) [32] and was conducted from February 2017 to August 2018.

### Study Setting and Sample

We enrolled patients with primary hypertension from a cardiovascular outpatient clinic of a medical center in northern Taiwan. The inclusion criteria were as follows: age of 20-79 years, diagnosis of primary hypertension with SBP≥130 mm Hg or DBP≥80 mm Hg, intake of less than four antihypertensive agents, access to a sphygmomanometer at home, ownership of a smart phone or personal computer to use, ability to read and understand Chinese or Taiwanese, and will to participate. The exclusion criteria were as follows: SBP≥180 mm Hg or DBP≥100 mm Hg; pregnancy; receipt of a heart transplant, permanent pacemaker, or implantable cardioverter defibrillator; diagnosis of arrhythmia, stroke, thyroid disease, major psychiatric disorder, renal disease, heart failure, acute myocardial infarction, cancer, or terminal disease; intake of antidepressants; or addiction to drugs or alcohol.

The study procedures were reviewed and approved by the Institutional Review Board (IRB 2-104-05-148) of the participating hospital. Before enrollment, the principal investigator explained the research purpose and procedures to the participants and obtained their written informed consent. The participants randomly assigned to the intervention group were trained to use the self-titration platform for 1 month and continuously received the self-titration intervention for 6 months. The participants in the control group received usual care.

### Study Procedures

The participants assigned to the intervention group received a 4-week training course before receiving the Web-based self-titration program. First, participants were given a secure account and a unique password of the website platform. We assisted participants with any set-up required on their smartphones or tablets. A stepwise instruction booklet was provided to guide log-in and use the platform. Second, the physicians of these participants set individualized blood pressure targets and explained the tailored medication titration instructions to each participant, who were then asked to rely on their home blood pressure recordings to titrate their medication doses. Third, participants were trained to measure their blood pressure by using automated electronic sphygmomanometers correctly. Finally, all participants received education about the management of hypertension.

When participants began the Web-based self-titration program, they were asked to measure their blood pressure before taking their medications and to report the data on the platform every day. We reviewed the data daily and provided a consultation through a phone call or website platform as needed for each individual participant. The physicians provided instructions to participants for any medication dosage change (increase or decrease), based on the self-monitoring data, through the website platform or clinical visit every month. The participants learned how to modify their lifestyle and manage hypertension by visiting the website repeatedly.

The participants in the control group received usual care, which included routine follow-up treatments for medication, lifestyle modification consultations, and a blood pressure check. Medications were adjusted depending on evaluations from their physicians at each clinical visit.

The principal investigator conducted a meeting to deal with problems arising in the study once a month. The outcomes were evaluated for both groups before starting the intervention and after initiating the intervention, at 3 months and 6 months.

### Intervention

**Figure 1:**
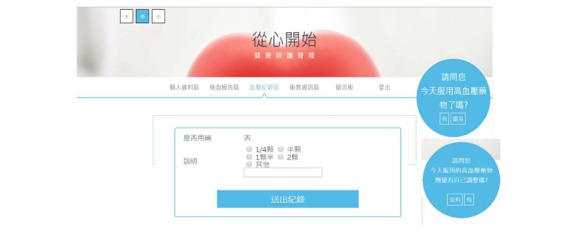
Screenshot of instruction in self-titration strategy.

Based on a review of the literature [33-35] and our professional experiences, our research team determined that patients able to titrate medications by themselves should be proficient in three areas: the ability to measure blood pressure correctly, the ability to record and understand the blood pressure recordings, and the ability to adjust their dose (add, maintain, or decrease) or adopt emergency interventions based on the instructions. A secure website was designed to assist patients in performing safe self-titration. The website includes five sections: (1) personal information collection, (2) individual physical data recordings, (3) blood pressure recordings, (4) patient education in hypertension, and (5) consultations (Figure 1). 

In the personal information section, we required patients to provide the following data: age, gender, education status, employment status, contact information, comorbidity, current medications, and next visit date. In the individual physical data section, we input hematology test data such as low-density lipoprotein cholesterol, high-density lipoprotein cholesterol, triglycerides, and serum creatinine levels through a chart review. Patients were able to access their blood test data through the website.

**Figure 2:**
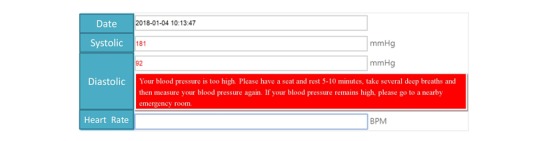
Screenshot of the medication reminder area and medication titration area.

**Figure 3:**
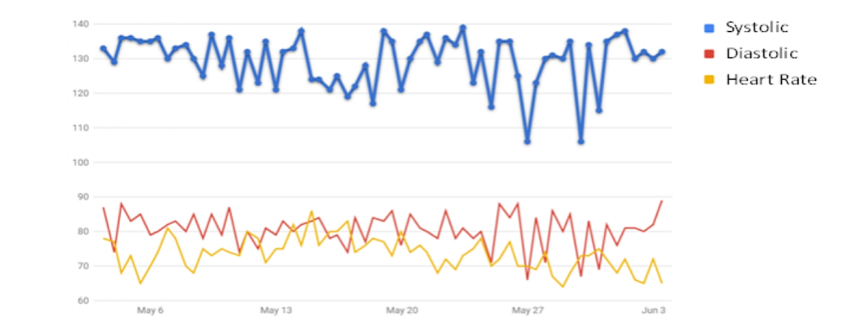
Screenshot of curve diagram on blood pressure.

In the blood pressure recording section, we set up individual blood pressure targets for each patient. The patients measured their blood pressure and recorded the data through the Web. An alarm and reminder system was designed and set up to allow patients to clearly understand the meaning of their current blood pressure readings and how to deal with them (Multimedia Appendix 1) [28,36]. When patients filled in their blood pressure data on the website, reminders popped up according to the blood pressure readings (Figure 2). If a blood pressure reading was below or above normal, the system automatically triggered an alert for the study team to contact the patient. If the blood pressure readings were not entered by 9 PM, the system was designed to send an email notification to remind the study team and either the patient or his/her caregiver to enter the readings. The database was automatically backed up at 12 PM each day. Outputs of blood pressure data were displayed as curve diagrams showing the 1-month or 3-month trends to patients (Figure 3).

In the patient education section, a video provided information about the management of hypertension and instructions about blood pressure measurements, a healthy diet, and exercise. The video content was designed based on the guidance of five experts from among the cardiologists and nurses. Finally, the patients were able to directly contact the research team through the consultation section.

### Measures

#### Demographic and Clinical Characteristics

The following data were collected from self-reports and chart review before randomization: age, gender, education, marital status, occupation, smoking habits, body mass index, clinical history, antihypertensive medications, and duration of having hypertension. We calculated the antihypertensive dosage based on the defined daily dose (DDD), as recommended by the WHO, which uses assumed average maintenance doses per day for a drug according to its main indication in adults [37].

#### Blood Pressure Measurements

Blood pressure was measured with an automatic sphygmomanometer (JPN1; Omron Colin, Kyoto, Japan). According to the guidelines of the Taiwan Society of Cardiology and the Taiwan Hypertension Society, we asked the participants to sit in a quiet and comfortable room for at least 5 minutes before taking their blood pressure. The blood pressure was taken twice at intervals of 1-2 minutes [38]. The average of these two readings was used in the data analysis. In addition, the researchers assisted all participants in checking the accuracy of their own automated electronic sphygmomanometers and evaluated whether the cuffs were appropriately sized for participants’ upper arms.

### Health-Related Quality of Life

We used the 3-level version of EuroQol five-dimension self-report questionnaire (EQ-5D-3L) [39] to measure variables of HRQoL. The EQ-5D-3L is composed of two parts: (1) the EQ-5D descriptive system and (2) the EuroQol visual analog scale (EQ-VAS) [39]. The EQ-5D descriptive system has five dimensions: mobility, self-care, usual activities, pain/discomfort, and anxiety/depression. Each dimension is rated on three levels: no problems (level 1), some problems (level 2), and extreme problems (level 3); the scores are converted into a single summary score, which describes a patient’s HRQoL. The EQ-VAS records the respondent’s self-rated health status on a 20-cm vertical scale (visual analog scale) that ranges from 0, indicating the worst health status, to 100, indicating the best possible health state [39]. The VAS is a quantitative measure of a patient’s judgement about their health status. We used the Chinese version of EQ-5D-3L, which has been demonstrated to have adequate validity and reliability [40].

### Defined Daily Dose

We calculated the antihypertensive dosage based on the DDD, as recommended by the WHO, a unit of measurement for assumed average maintenance doses per day for a drug according to its main indication in adults. The DDD provides a standardized and objective dose unit, allowing clinicians or researchers to assess drug consumption dosage and compare patients with themselves or other patients. DDDs are only used for medicines after they are assigned Anatomical Therapeutic Chemical Classification System (ATC) codes [37]. For example, the ATC code for amlodipine (5 mg) is C08CA01. This code indicates that the DDD of amlodipine is 5 mg. Therefore, when the prescription of daily dose of amlodipine is 5 mg, the patient is taking 1 DDD of this medicine [37].

### Outcomes

The primary outcome was the mean SBP and DBP at 3 months and 6 months. The secondary outcomes were the overall antihypertensive DDD and the two measures for HRQoL at 3 months and 6 months.

### Statistical Analysis

We estimated the sample size using G*Power version 3.1 [41]. According to a previous study [28], a sample size of 80 patients per group was estimated for 90% power (two-tailed and at a 5% significance level) to detect a difference of at least 5 mm Hg in systolic blood pressure between the intervention and control groups and assuming an SD of 17 mm Hg. Allowing for a 20% dropout rate during follow-up, the sample size was increased to at least 96 patients for each group.

Data were analyzed by IBM SPSS, Version 21.0 (IBM Corp, Armonk, New York) based on intention-to-treat analysis. The analysis was conducted by a researcher who was blinded to the random allocation. The demographic and clinical characteristics were analyzed as means and SDs or as frequencies and percentages. Independent *t*-tests and Chi-square tests were carried out to examine the homogeneity of characteristics between the two groups. Group differences were examined using the generalized estimating equation (GEE). The outcomes were tested at the 5% two-tailed level of significance.

## Results

### Baseline Participant Characteristics

**Figure 4:**
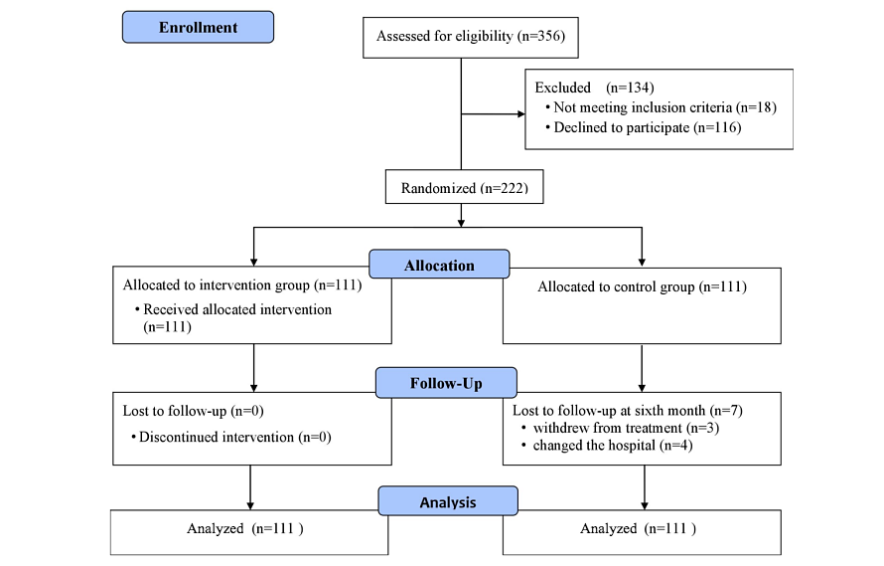
Flow diagram of the inclusion of patients in the randomized trial of the two groups.

Of the 356 enrolled patients, 18 did not meet the inclusion criteria and 116 (32.6%) declined to participate. Therefore, a total of 222 patients (62.4%) were included and randomly assigned to the intervention (n=111) or control (n=111) group. Figure 4 shows the flowchart for screening, enrollment, randomization, and follow-up.

The baseline characteristics of participants are summarized in Multimedia Appendix 2. The mean age of participants was 62.7 (SD 9.3) years. Most participants were men (n=114, 51%), married (n=214, 94%), and employed (n=124, 56%); only a few participants were smokers (n=28, 13%). The mean body mass index was 26.44 kg/m^2^ (SD 3.79). The average length of hypertension diagnosis was approximately 6 years, and diabetes and hyperlipidemia were the most common comorbidities. The mean SBP and DBP were 143.21 (SD 13.62) mm Hg and 84.18 (SD 10.84) mm Hg, respectively. The overall antihypertensive DDD was 1.80 (SD 1.0) units. Only the demographic characteristic of education differed between groups (*P*<.001); however, there were no significant differences in any clinical characteristics.

### Effect on Systolic Blood Pressure

We used GEE analysis to examine if there was an effect of the Web-based self-titration program on the control of SBP (Table 1). After adjusting for the baseline difference in education, the GEE model showed that the SBP decreased significantly more for the intervention group than for the control group at the 3- and 6-month follow-ups, as shown in Multimedia Appendix 3. The findings indicated that the Web-based self-titration program resulted in a greater control of participants’ SBP.

**Table 1:** Generalized estimating equation analysis of the effect of the intervention on systolic blood pressure.

Variable	Regression coefficient	SE	χ^2^ (df)	*P* value
Group (intervention)^a^	0.12	1.86	0.004 (1)	.95
Time (3 mo)^b^	5.28	1.36	15.2 (1)	<.001
Time (6 mo)^b^	7.99	1.44	30.9 (1)	<.001
Group (intervention) × time (3 mo)^c^	–21.43	1.89	127.7 (1)	<.001
Group (intervention) × time (6 mo)^c^	–27.82	2.10	175.2 (1)	<.001

^a^Reference group: control group.

^b^Reference group: time (baseline).

^c^Reference group: group (control) × time (baseline).

### Effect on Diastolic Blood Pressure

GEE analysis examined changes in DBP after controlling for the difference between groups at baseline. The mean DBP for the intervention group significantly decreased more at 3- and 6-month follow-ups (*P*<.001) compared to the control group (Table 2; Multimedia Appendix 4). These findings suggest that patients who participated in the Web-based self-titration program had better control of DBP than patients in the control group who received usual care.

**Table 2:** Generalized estimating equation analysis of the effect of the intervention on diastolic blood pressure.

Variable	Regression coefficient	SE	χ^2^ (df)	*P* value
Group (intervention)^a^	0.536	1.44	0.1 (1)	.71
Time (3 mo)^b^	–1.586	0.83	3.6 (1)	.057
Time (6 mo)^b^	0.477	1.03	0.2 (1)	.64
Group (intervention) × time (3 mo)^c^	–5.442	1.21	20.4 (1)	<.001
Group (intervention) × time (6 mo)^c^	–9.739	1.49	42.6 (1)	<.001

^a^Reference group: control group.

^b^Reference group: time (baseline).

^c^Reference group: group (control) × time (baseline).

### Effect on Overall Defined Daily Dose for Antihypertensive Medication

GEE analysis examined the effect of the Web-based self-titration program on changes in overall DDD for antihypertensive medicines. The GEE model, adjusted for the baseline difference between groups, showed that the mean DDD for the intervention group significantly decreased more than that for the control group (*P*<.01) at both follow-up times (Table 3; Multimedia Appendix 5). Our findings indicate that patients who participated in the Web-based self-titration program intervention were able to significantly reduce the dosage of antihypertensive medicine.

**Table 3:** Generalized estimating equation analysis of the effect of the intervention on overall defined daily dose.

Variable	Regression coefficient	SE	χ^2^ (df)	*P* value
Group (intervention)^a^	–0.242	0.16	2.5 (1)	.12
Time (3 mo)^b^	0.132	0.47	8.1 (1)	.004
Time (6 mo)^b^	0.132	0.05	6.9 (1)	.008
Group (intervention) × time (3 mo)^c^	–0.202	0.07	8.9 (1)	.003
Group (intervention) × time (6 mo)^c^	–0.236	0.07	11.3 (1)	.001

^a^Reference group: control group.

^b^Reference group: time (baseline).

^c^Reference group: group (control) × time (baseline).

### Effect on Health-Related Quality of Life

GEE analysis of EQ-5D scores examined the effect of the Web-based self-titration program on improving HRQoL (Table 4). After adjusting for the baseline difference between groups, the GEE model showed that the increase of the mean EQ-5D scores for the intervention group was significantly more (*P*<.001) than that for the control group at the 3- and 6-month follow-ups (Table 4; Multimedia Appendix 6). These findings indicate that patients who were provided with the Web-based self-titration program perceived their HRQoL to improve following the intervention and compared to controls who did not participate in the program.

**Table 4:** Generalized estimating equation analysis of the effect of the intervention on EuroQol five-dimension self-report questionnaire scores.

Variable	Regression coefficient	SE	χ^2^ (df)	*P* value
Group (intervention)^a^	–0.074	0.02	17.5 (1)	<.001
Time (3 mo)^b^	–0.076	0.02	23.7 (1)	<.001
Time (6 mo)^b^	–0.108	0.02	40.7 (1)	<.001
Group (intervention) × time (3 mo)^c^	0.216	0.02	113.1 (1)	<.001
Group (intervention) × time (6 mo)^c^	0.275	0.02	171.6 (1)	<.001

^a^Reference group: control group.

^b^Reference group: time (baseline).

^c^Reference group: group (control) × time (baseline).

### Effect on Self-Rated Health Status

The EQ-VAS used to measure participants’ self-rated health status was also evaluated at the 3- and 6-month follow-ups using the GEE model adjusted for the baseline difference between groups (Table 5). The mean scores on the EQ-VAS increased significantly more for the intervention group than the control group at both follow-up times (*P*<.001) (Table 5; Multimedia Appendix 7). Thus, these findings suggest that the Web-based self-titration intervention resulted in improvement in perception of patients’ health status at the 3- and 6-month follow-ups.

**Table 5:** Generalized estimating equation analysis of the effect of the intervention on self-rated health status.

Variable	Regression coefficient	SE	χ^2^ (df)	*P* value
Group (intervention)^a^	–11.247	1.89	35.5 (1)	<.001
Time (3 mo)^b^	–8.252	1.56	28.1 (1)	<.001
Time (6 mo)^b^	–12.820	1.65	60.5 (1)	<.001
Group (intervention) × time (3 mo)^c^	24.459	1.92	163.0 (1)	<.001
Group (intervention) × time (6 mo)^c^	36.883	2.08	314.4 (1)	<.001

^a^Reference group: control group.

^b^Reference group: time (baseline).

^c^Reference group: group (control) × time (baseline).

## Discussion

### Principal Findings

This randomized clinical trial examined the effects of a web-based self-titration program on the control of blood pressure and HRQoL in patients with primary hypertension. The principal findings of this trial demonstrated that our Web-based self-titration program significantly improved blood pressure control, overall DDD for antihypertensive medicine, HRQoL, and self-rated health status at 3- and 6-month follow-ups compared to baseline measures. In addition, these improvements were significantly better than those seen in the control group at both follow-ups. Additionally, no harmful events occurred in our cohort.

Our results on the control of SBP and DBP are consistent with the findings of previous studies on the benefits of self-titration of antihypertensive medication for blood pressure control [27,28]. In our study, we designed a program to assist patients with hypertension to self-monitor their blood pressure in their home every day; self-manage their life style; and, when needed, self-titrate their antihypertensive medication through an internet website. The self-monitoring of blood pressure has been identified as an effective means of blood pressure control [18]. Moreover, a meta-analysis of 25 clinical trials suggested the implementation of self-monitoring in conjunction with co-interventions, such as lifestyle counselling and systematic medication titration by doctors, pharmacists, or patients, leads to better clinical blood pressure control for patients with hypertension [20]. Our Web-based intervention program resulted in significant blood pressure reduction in patients with hypertension. Our findings provide further support that daily patient self-management of treatment and lifestyle through a website can lead to better control of blood pressure for patients with hypertension.

Another notable finding of our study was that the overall antihypertensive DDD was significantly reduced in the intervention group compared with the control group. No other studies have reported similar findings. The significant reduction in antihypertensive DDD for patients in the intervention group may have been a result of consultations and reminders from clinical professionals through the Web-based program. This support may have helped patients not only modify their lifestyle, but also persist in carrying out healthy behavior. Empowerment for lifestyle self-management and self-titration of their own medication has been shown to positively impact patient compliance [27]. Therefore, a more positive attitude of commitment to these aspects could have been promoted by an increased sense of autonomy and control over the disease. Regardless of the reason, this finding appears to be in line with international guidance that adherence to both lifestyle change and medication are equally important in blood pressure control [20].

HRQoL is frequently used to examine the beneficial effects of interventions and treatments [13]. Earlier studies in patients with hypertension indicated that poor blood pressure control negatively affected HRQoL [13-15]. Hypertensive patients with poor blood pressure control have greater stresses resulting from the disease itself and the treatments, and therefore, they may have worse HRQoL as a result of coping with related stress [13,42]. In this study, we found that patients with hypertension in the intervention group who received the Web-based self-titration program had greater improvement in their HRQoL at the 3- and 6-month follow-ups compared with the patients in the control group. We suggest that control of blood pressure for patients with hypertension in the intervention program helped in improving their quality of life. The patients in the intervention group reported that the Web-based self-titration program led them to engage in monitoring their blood pressure more regularly and empowered them to self-manage their clinical interventions and lifestyle. The educational component of the intervention provided patients with knowledge about the relationship between blood pressure and health status. They felt that they benefited from the support offered through the online consultations, where they could communicate problems with blood pressure control immediately. Therefore, they felt more confident that they may control their blood pressure and life.

Our results also showed that patients who engaged in the Web-based self-titration program perceived a significant improvement in their health status at the 3- and 6-month follow-ups. Health status is challenging to improve because it is a patient-centered outcome. The symptom burden significantly predicts worse health status [43]. In this study, patients were empowered to self-manage their blood pressure and achieved a positive outcome. The reduction in symptoms, owing to the control of their blood pressure, may have improved their perceived health status. In addition to symptom burden, HRQoL is another principal component of health status [44]. The hypertensive patients who received the Web-based self-titration program reported a significant improvement in their HRQoL, which may have also advanced in their health status.

### Limitations

The potential limitations in this study should be considered. First, our follow-up period was only 6 months, which meant that we could not detect the long-term consequences on cardiovascular events, or indeed, whether the initial successes would be sustainable in the long term. Nevertheless, the initial improvements in blood pressure control have a clear potential to reduce cardiovascular complication rates. Second, we used telemedicine to support the medication titration, which excluded patients without a computer or smartphone access. Third, we were not able to blind the patients’ physicians, which could have introduced bias. Finally, we only recruited participants from one medical center, which limits our ability to generalize the findings. These limitations should be addressed in future research.

### Conclusions

There is limited research examining the effects of a Web-based self-titration program on blood pressure control in patients with primary hypertension. The results of this study support both our hypotheses: (1) patients with primary hypertension who received the Web-based self-titration program had significant control of SBP and DBP, and (2) the HRQoL of patients was significantly improved through this Web-based program. In addition, the intervention group had significant reductions in DDD for antihypertensive medications and improvements in the perception of their health status. Thus, we believe that the Web-based self-titration program may assist patients with primary hypertension to self-manage their treatments and healthy lifestyle in their home. This Web-based intervention program also has the benefit of reducing the amount of time required for patients to visit an outpatient clinic or hospital for care. Taken together, the intervention program could improve the quality of care for patients while reducing health care costs.

## Appendix

Multimedia Appendix 1 Alarm and Reminder system: Definitions of blood pressure readings and actions.

Multimedia Appendix 2 Demographic and clinical characteristics of participants and differences between groups.

Multimedia Appendix 3 Change in systolic blood pressure between two groups at 3 times.

Multimedia Appendix 4 Change in diastolic blood pressure between two groups at 3 times.

Multimedia Appendix 5 Change in the overall antihypertensive defined daily dose between two groups at 3 times.

Multimedia Appendix 6 Change in scores of EQ5D between two groups at 3 times.

Multimedia Appendix 7 Change in scores of ED-VAS between two groups at 3 times.

Multimedia Appendix 8 CONSORT‐EHEALTH checklist (V 1.6.1).

## Abbreviations

DBP: diastolic blood pressure
DDD: defined daily dose
EQ-5D-3L: 3-level version of EuroQol five-dimension self-report questionnaire
EQ-VAS: EuroQol visual analogue scale
GEE: generalized estimating equation
HRQoL: health-related quality of life
SBP: systolic blood pressure
WHO: World Health Organization
