# Supplementary material for: A Web-Based Self-Titration Program to Control Blood Pressure in Patients With Primary Hypertension: Randomized Controlled Trial
Source: J Med Internet Res. 2019 Dec 5;21(12):e15836. doi: 10.2196/15836 (PMC6923762; doi:10.2196/15836)
Supplement: Multimedia Appendix 1 [file jmir_v21i12e15836_app1.docx]

**Table1.** Alarm and Reminder system: Definitions of blood pressure readings and actions

| Color | Level | Blood Pressure | Action |
| --- | --- | --- | --- |
|  |  |  |  |
| Red | High | SBP ≥ 180  Or  DBP ≥ 110 | - Your blood pressure is too high. - Please have a seat and rest 5–10 minutes, take several deep breaths, and then measure your blood pressure again. - If your blood pressure remains high, please go to a nearby emergency room. |
| Yellow | Raised | SBP = 131-179  Or  DBP = 81-109 | - Your blood pressure is raised. You need to pay attention to your medication. - Please have a seat and rest 5–10 minutes, take several deep breaths, and then measure your blood pressure again. - If your blood pressure remains high, or you are still feeling uncomfortable, you can look at your medication adjustment instructions or consult us. |
| Green | Normal | SBP = 90–130  and  DBP ≤ 80 | - Your blood pressure is normal. - Please keep measuring and recording your blood pressure. - Please keep monitoring side effects of your medication. - If you are feeling uncomfortable, please make an appointment within 72 hours with your physician. |
| Blue | Low | SBP ≤ 89 | - Your blood pressure is too low. - Please lie in bed 5-19 minutes, and then measure your blood pressure again. - If your blood pressure remains low, or you are still feeling uncomfortable (e.g., dizziness, lightheadedness on standing, etc.), please go to a nearby emergency room. |
