# Supplementary material for: A Web-Based Self-Titration Program to Control Blood Pressure in Patients With Primary Hypertension: Randomized Controlled Trial
Source: J Med Internet Res. 2019 Dec 5;21(12):e15836. doi: 10.2196/15836 (PMC6923762; doi:10.2196/15836)
Supplement: Multimedia Appendix 2 [file jmir_v21i12e15836_app2.docx]

**Table2.** Demographic and clinical characteristics of participants and differences between groups

| Variable | Total  (N = 222) | Intervention  (n = 111) | Control  (n = 111) | χ^2^ / t | *p* |
| --- | --- | --- | --- | --- | --- |
| Age, mean (SD), y | 62.73 (9.30) | 62.07 (9.77) | 63.40 (8.80) | -1.06 | 0.29 |
| Gender, n (%) |  |  |  | 0.07 | 0.89 |
| Male, n (%) | 114 (51.4) | 56 (50.5) | 58 (52.3) |  |  |
| Employment status |  |  |  | -0.07 | 0.79 |
| Employed, n (%) | 124 (55.9) | 63 (56.8) | 61 (55.0) |  |  |
| Education, n (%) |  |  |  | 29.48 | < .001 |
| Uneducated | 13 (5.9) | 4 (3.6) | 9 (8.1) |  |  |
| Elementary school | 46 (20.7) | 10 (9.0) | 36 (32.4) |  |  |
| Junior high school | 34 (15.3) | 14 (12.6) | 20 (18.0) |  |  |
| Senior high school | 45 (20.3) | 26 (23.4) | 19 (17.1) |  |  |
| University/college | 84 (37.8) | 57 (51.4) | 27 (24.3) |  |  |
| Marital status, n (%) |  |  |  | 3.15 | 0.21 |
| Single | 5 (2.3) | 2 (1.8) | 3 (2.7) |  |  |
| Married | 208 (93.7) | 107 (96.4) | 101 (91.0) |  |  |
| Divorced | 9 (4.1) | 2 (1.8) | 7 (6.3) |  |  |
| Smoker, n (%) | 28 (12.6) | 15 (13.5) | 13 (11.7) | 0.16 | 0.84 |
| BMI, mean (SD) | 26.44 (3.79) | 26.38 (3.73) | 26.50 (3.87) | -0.23 | 0.82 |
| Duration of hypertension mean (SD), mo | 73.25 (62.11) | 72.12 (67.46) | 74.34 (56.54) | -0.26 | 0.80 |
| DDD, mean (SD) | 1.80 (1.00) | 1.68 (0.85) | 1.92 (1.14) | 0.71 | 0.48 |
| Comorbidity, n (%) |  |  |  |  |  |
| CAD | 41 (18.5) | 17 (15.3) | 24 (21.6) | 1.47 | 0.23 |
| Diabetes | 85 (38.3) | 40 (36.0) | 45 (40.5) | 0.48 | 0.49 |
| Hyperlipidemia | 105 (47.3) | 50 (45.0) | 55 (49.5) | 0.45 | 0.50 |
| Gout | 23 (10.4) | 9 (8.1) | 14 (12.6) | 1.21 | 0.27 |
| SBP, mean (SD), (mmHg) | 143.21 (13.62) | 142.96 (14.12) | 143.45 (13.17) | -0.27 | 0.79 |
| DBP, mean (SD), (mmHg) | 84.18 (10.84) | 84.41 (10.83) | 83.95 (10.91) | 0.31 | 0.76 |

Abbreviations: SD, standard deviation; BMI, body mass index; CAD, coronary artery disease; DBP, diastolic blood pressure; DDD = Defined daily dose (for antihypertensive); SBP, systolic blood pressure
