# Supplementary figures and images for: A Web-Based Self-Titration Program to Control Blood Pressure in Patients With Primary Hypertension: Randomized Controlled Trial
Source: J Med Internet Res. 2019 Dec 5;21(12):e15836. doi: 10.2196/15836 (PMC6923762; doi:10.2196/15836)

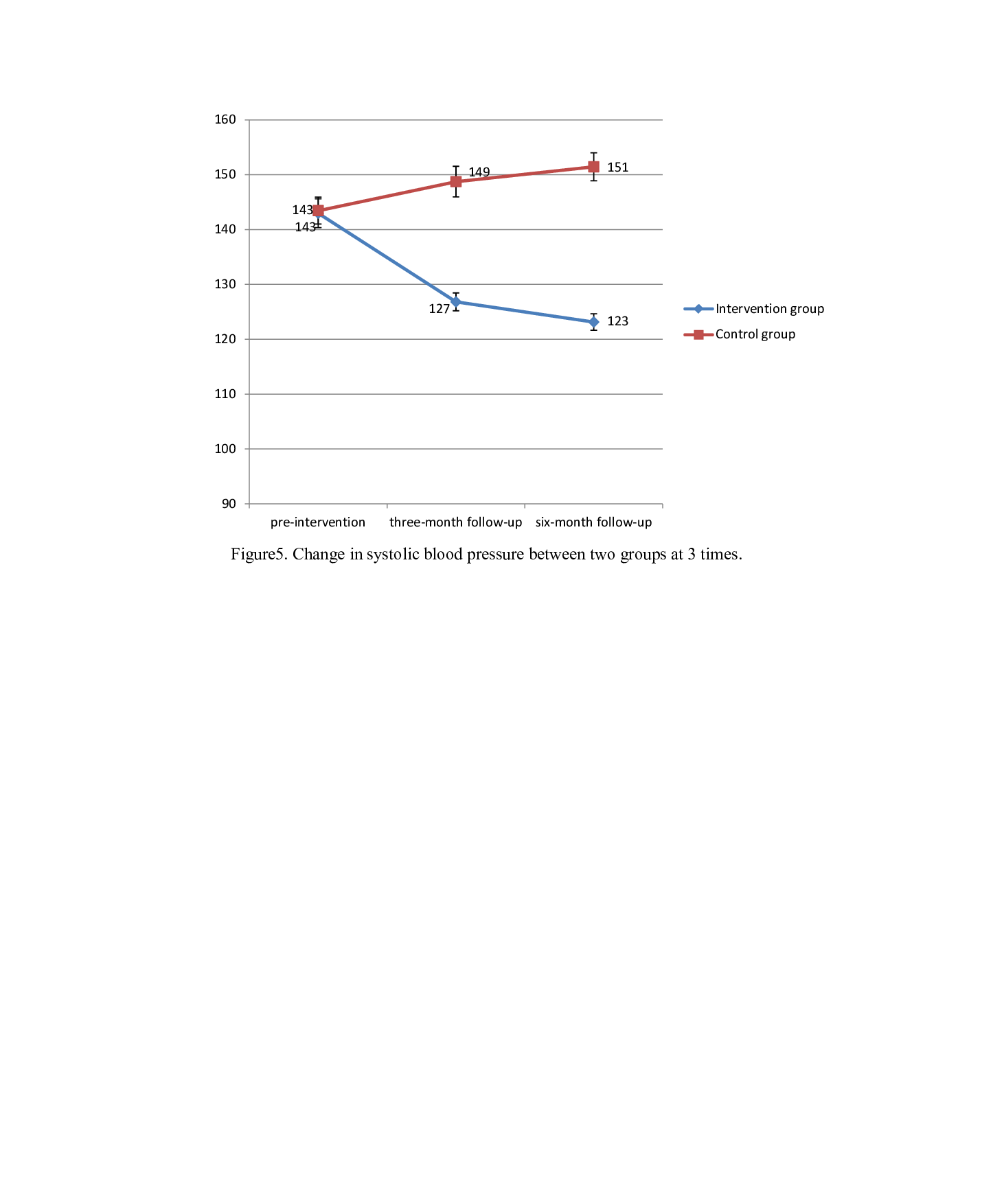

Supplement: Multimedia Appendix 3 [file jmir_v21i12e15836_app3.png]

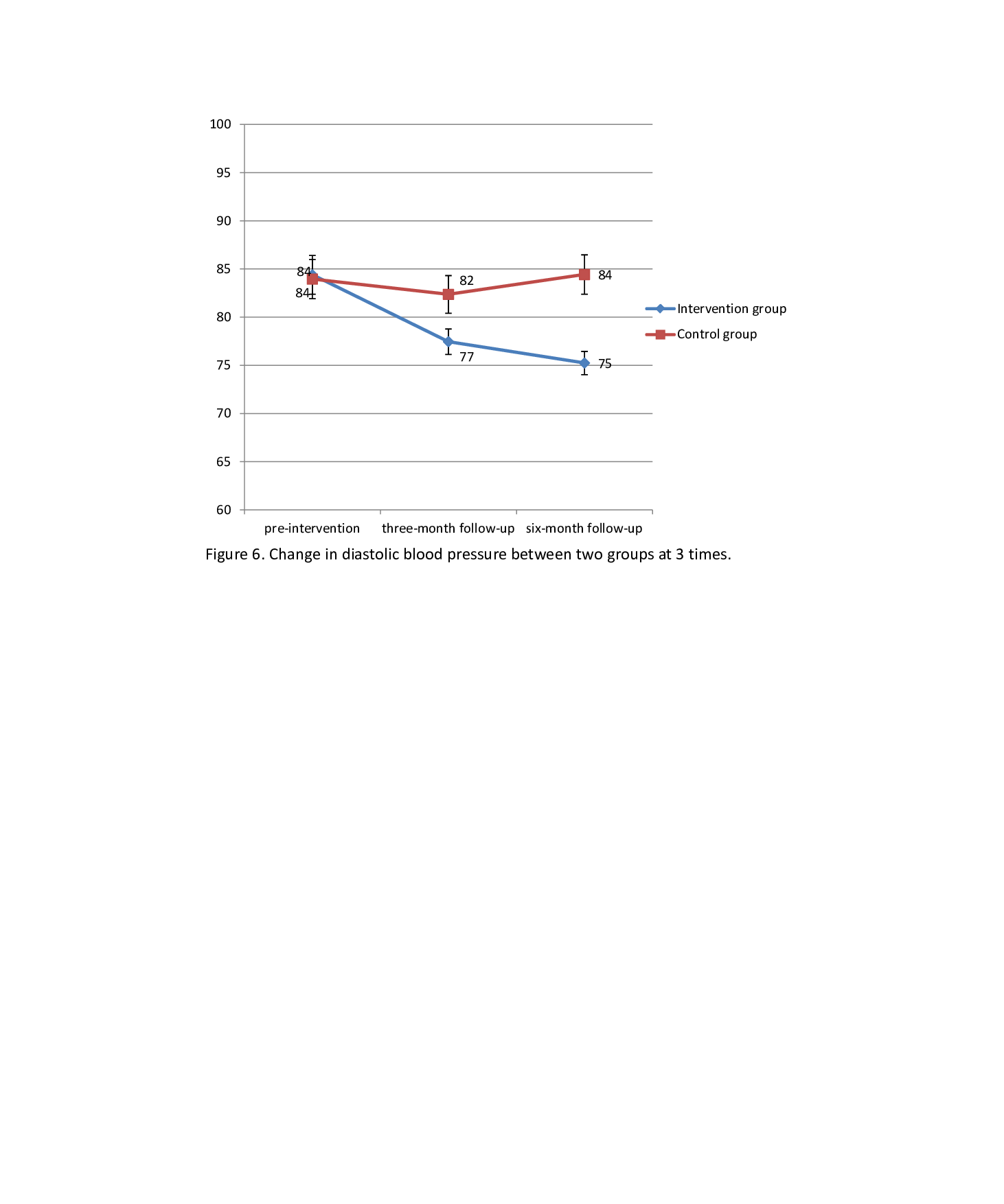

Supplement: Multimedia Appendix 4 [file jmir_v21i12e15836_app4.png]

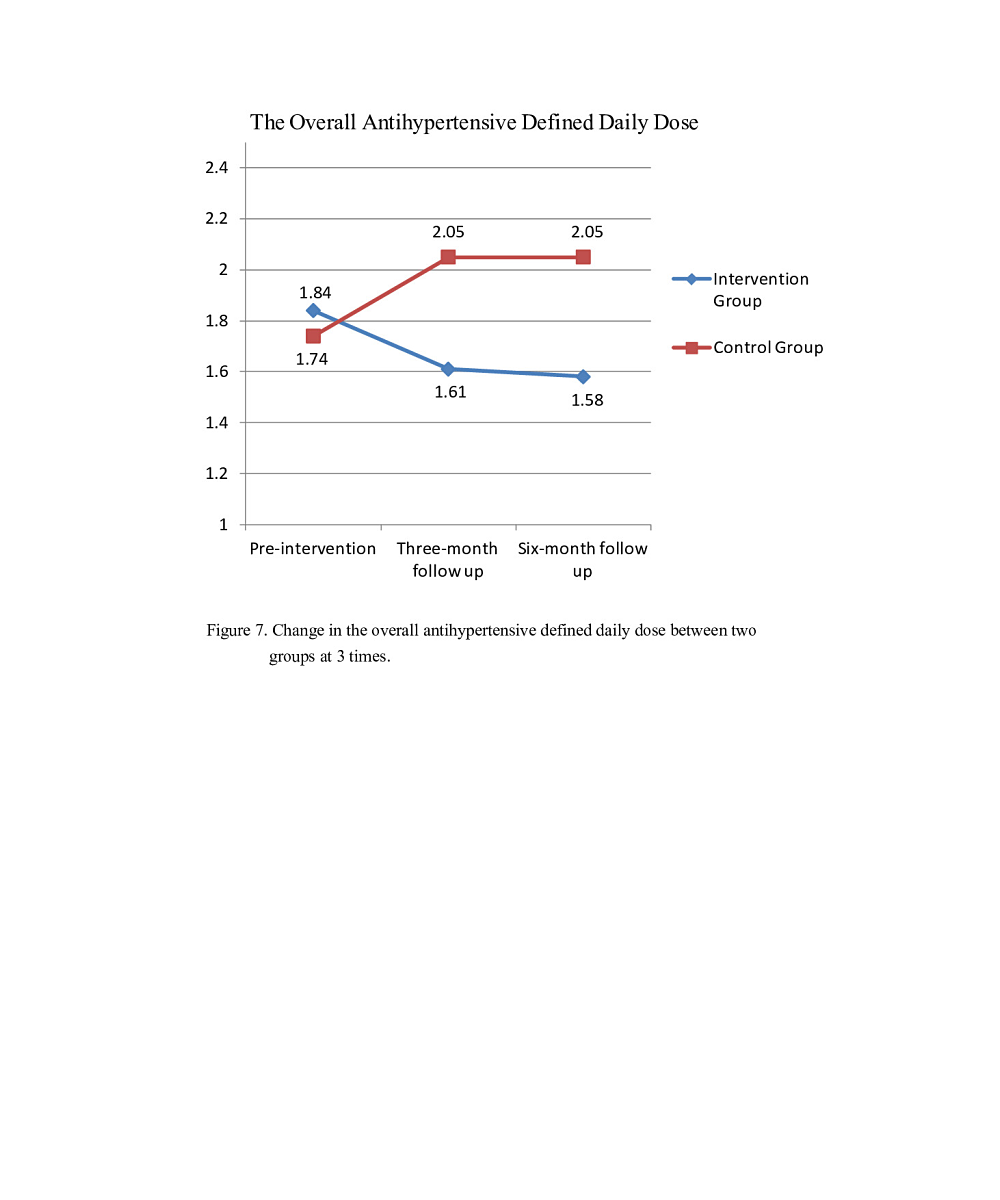

Supplement: Multimedia Appendix 5 [file jmir_v21i12e15836_app5.png]

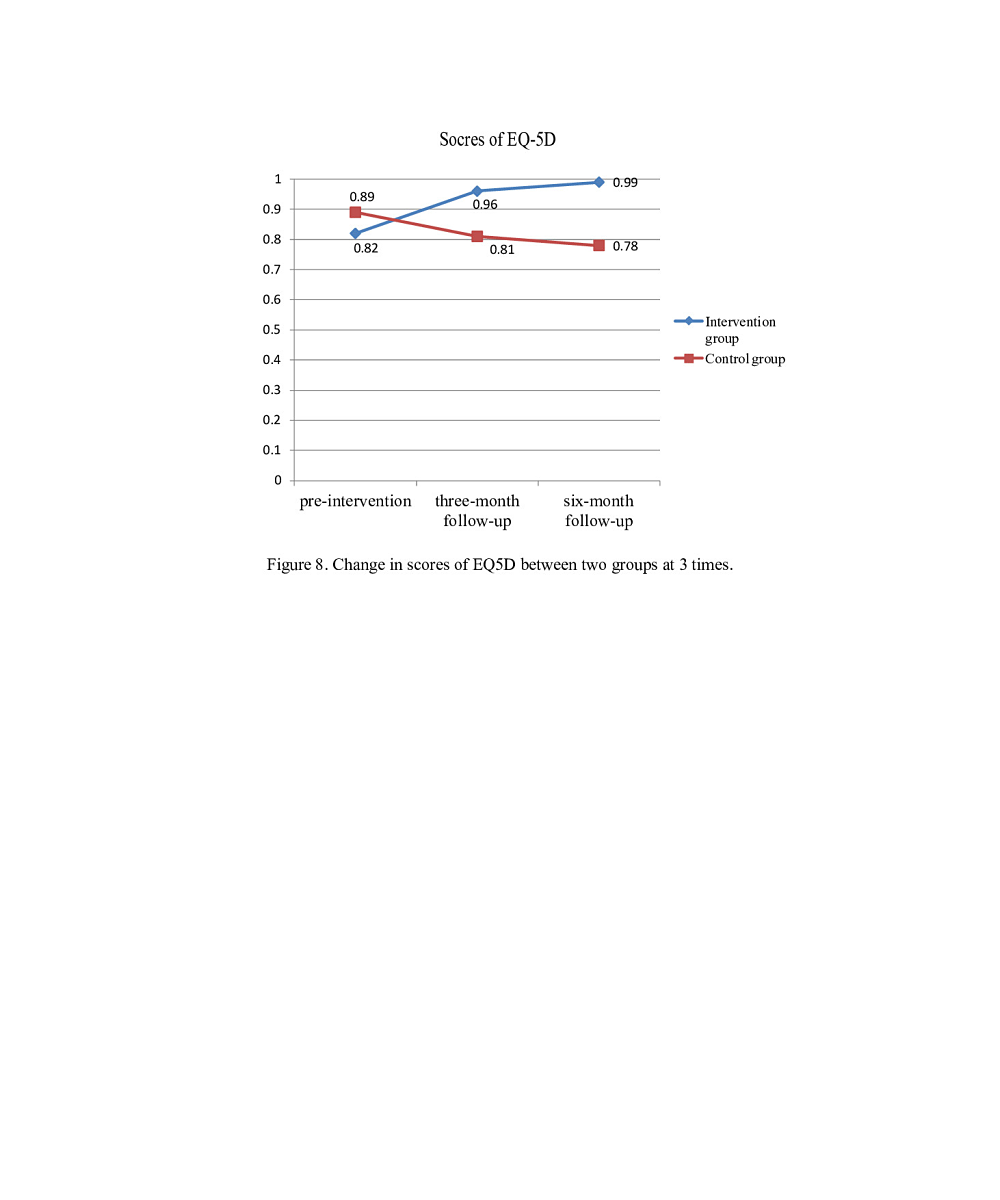

Supplement: Multimedia Appendix 6 [file jmir_v21i12e15836_app6.png]

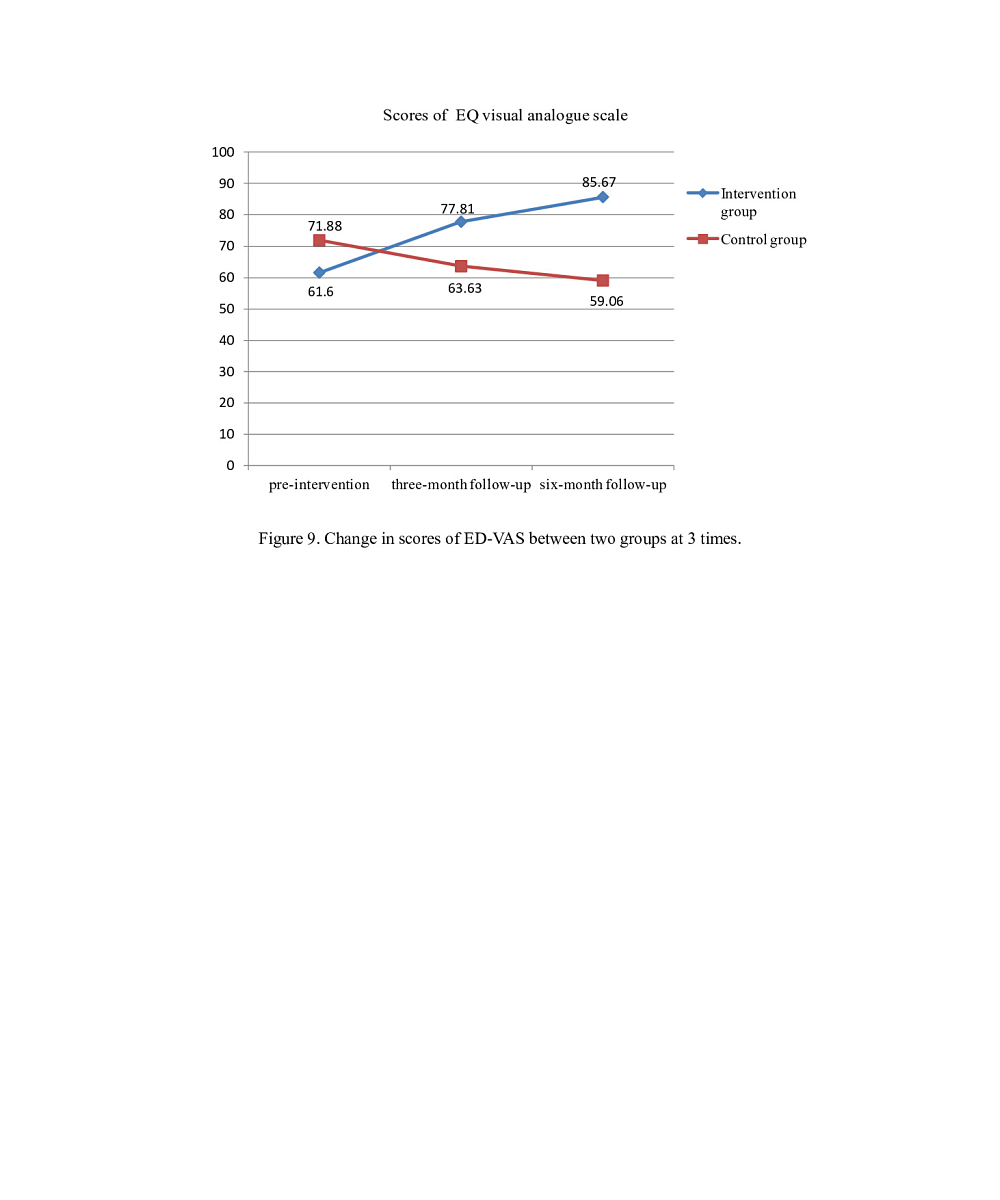

Supplement: Multimedia Appendix 7 [file jmir_v21i12e15836_app7.png]
